# Lung Ultrasonography in the Monitoring of Intraoperative Recruitment Maneuvers

**DOI:** 10.3390/diagnostics11020276

**Published:** 2021-02-10

**Authors:** Jolanta Cylwik, Natalia Buda

**Affiliations:** 1 Anesthesiology and Intensive Care Unit, Mazovia Regional Hospital, 08-110 Siedlce, Poland; jolacylwik@o2.pl; 2 Department of Internal Medicine, Connective Tissue Diseases and Geriatrics, Medical University of Gdansk, 80-210 Gdańsk, Poland

**Keywords:** atelectasis, intensive care, respiratory failure, chest ultrasonography

## Abstract

Introduction: Postoperative respiratory failure is a serious problem in patients who undergo general anesthesia. Approximately 90% of mechanically ventilated patients during the surgery may develop atelectasis that leads to perioperative complications. Aim: The aim of this study is to determine whether it is possible to optimize recruitment maneuvers with the use of chest ultrasonography, thus limiting the risk of respiratory complications in patients who undergo general anesthesia. Methodology: The method of incremental increases in positive end-expiratory pressure (PEEP) values with simultaneous continuous ultrasound assessments was employed in mechanically ventilated patients. Results: The study group comprised 100 patients. The employed method allowed for atelectasis reduction in 91.9% of patients. The PEEP necessary to reverse areas of atelectasis averaged 17cmH_2_O, with an average peak pressure of 29cmH_2_O. The average PEEP that prevented repeat atelectasis was 9cmH_2_O. A significant improvement in lung compliance and saturation was obtained. Conclusions: Ultrasound-guided recruitment maneuvers facilitate the patient-based adjustment of the process. Consequently, the reduction in ventilation pressures necessary to aerate intraoperative atelectasis is possible, with the simultaneous reduction in the risk of procedure-related complications.

## 1. Introduction

Despite extensive advances in regional anesthesia methods, general anesthesia still remains indispensable for some surgical procedures—approximately 90% of surgical patients develop disturbances of lung aeration following positive pressure mechanical ventilation [1,2]. The risk of perioperative atelectasis depends on many factors, such as the type and duration of surgery, surgical technique and patient’s general status (obesity, comorbidities). The manner of anesthesia administration and mechanical ventilation is also of significance [3,4,5]. The extent and severity of perioperative disturbances of lung aeration may vary from small and clinically insignificant local hypoventilation areas to the appearance of large areas of completely nonaerated lung tissue. This may contribute to the development of intra- and post-operative complications [4,6], including gas exchange pathologies (mainly hypoxia), and may potentially trigger a local inflammatory response leading to lung damage (VILI—ventilator-induced lung injury) [7,8,9]. Recruitment maneuvers are a routine procedure for reducing aeration disturbances. Many recruitment techniques have been described in the literature [10,11,12,13,14]. However, irrespective of how the recruitment is administered, this procedure involves the risk of complications (e.g., barotrauma, volutrauma, hemodynamic destabilization). 

Lung ultrasonography is a modality facilitating bedside, quick and accurate diagnosis of atelectasis during general anesthesia [15,16,17,18,19]. The major advantage of ultrasound assessment is that it can be done in the operating room, repetitively and noninvasively, without the necessity to transport the patient to the radiology unit. 

The aim of the study is to determine whether the application of chest ultrasonography allows optimizing intraoperative recruitment maneuvers that reduce atelectasis in mechanically ventilated patients under general anesthesia. The question was asked whether it is possible to determine patient-specific pressure necessary to open collapsed alveoli and pressure that prevents repeat alveolar collapse, and further whether such a procedure allows reducing pressure during recruitment maneuvers (as compared to traditional methods), thus increasing the safety of the procedure both in the context of hemodynamic disturbances and the development of volutrauma.

## 2. Material and Methods 

### 2.1. Ethic Statement

The study was approved by the Bioethical Committee of the Regional Medical Chamber in Warsaw (no KB/1154/19, approval date 19 September 2019).

### 2.2. Patient Qualification 

Adult patients undergoing general anesthesia during elective and emergency surgery, who were able to provide their written informed consent for the participation in the study were qualified. They were assessed as ASA1, ASA2, ASA3 or ASA4 according to the ASA score (the American Society of Anesthesiologists physical status classification system). Exclusion criteria included: age below 18 years, risk of ASA5, pregnancy, patients with increased intracranial pressure and the inability to provide conscious informed consent for participation in the study. Patients undergoing chest surgeries were also excluded. 

### 2.3. Ultrasound Technique and Settings

**Figure 1:**
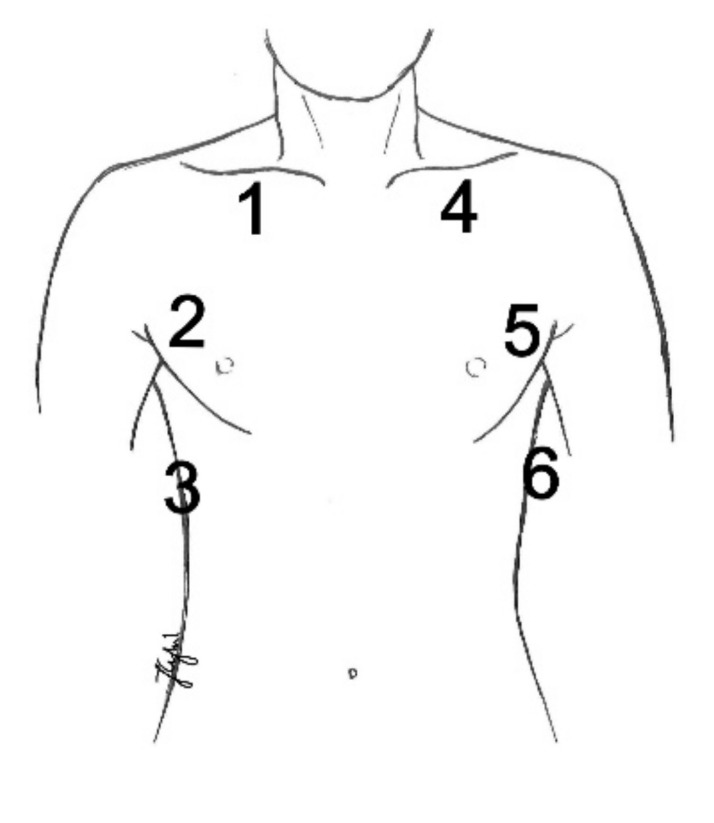
Lung ultrasound assessment points in the study.

Ultrasound examinations were performed and recorded with Philips Sparq ultrasound unit (Philips, Bothell, WA, USA), with a convex transducer (2–6 MHz) and linear transducer (5–12 MHz). The type of transducer was individually selected depending on the patient’s constitution. The examination was performed with the LUNG preset (characterized by speckle reduction, compound imaging, and tissue harmonic imaging filters switched off). The examination was performed by one anesthesiologist, with 10 years’ experience in lung ultrasonography. Patients were always examined in the supine position. The transducer head was applied at 6 points over the anterior and lateral part of the chest, symmetrically at 3 assessment points on each side and evaluated repeatedly. The first point was localized subclavically in the midclavicular line; the second point was located at the level of the 4th intercostal space in the anterior axillary line, and the third point was in the posterior axillary line at the level of the costodiaphragmatic recess (Figure 1).

The transducer head was preferably placed along the intercostal space to visualize the longest possible section of the pleura. The obtained image was qualified to a specific group (A profile—normal image, B profile—presence of B-line artifacts typical of the interstitial syndrome, C profile—subpleural consolidations characteristic for atelectasis, P profile—pleural effusion), and the result was recorded in the examination protocol, providing also current mechanical ventilation parameters and transcutaneous oxygen saturation levels (SaO_2_). 

### 2.4. Initial Mechanical Ventilation Parameters 

All patients qualified for the study were monitored and administered with general anesthesia depending on age, comorbidities and the extent of surgery. After intubation, mechanical ventilation was performed in the volume control ventilation (VCV) mode in a uniform manner (Philips IntelliSave AX700 anesthesia machine). Tidal volume (Vt) was set at 7 mL/kg of body mass, and the frequency of breaths was regulated so that end-tidal CO_2_ (EtCO_2_) was at the level of 35–40 mm Hg. Additionally, FiO_2_ was 0.35, and the initial positive end-expiratory pressure (PEEP) was always 5cmH_2_O.

### 2.5. Intraoperative Ultrasound Assessment Protocol

The first ultrasound assessment was performed before the induction of general anesthesia, and the second 10 min after intubation and the beginning of mechanical ventilation. When features of atelectasis were detected during the first or second assessment, the patient was qualified for the recruitment maneuver to be administered as quickly as possible. When ultrasonographic features of atelectasis were absent during the initial assessment, depending on the patient’s status during anesthesia and the results of taken measurements (transcutaneous blood gas monitoring, lung compliance), the decision concerning ultrasound reassessment was made. It was assumed that the decrease in transcutaneous oxygen saturation (SaO_2_) below 94% or decrease in lung compliance by a minimum of 15% would indicate the necessity of repeated lung assessment for atelectasis (compliance determined automatically by the anesthesia machine). 

When areas of atelectasis were visualized in the lungs, having excluded contraindications, the recruitment algorithm was introduced entirely guided by ultrasound. The patient’s hemodynamic instability was the contraindication for the maneuver [20,21].

### 2.6. Recruitment Protocol in the Study Group 

**Figure 2:**
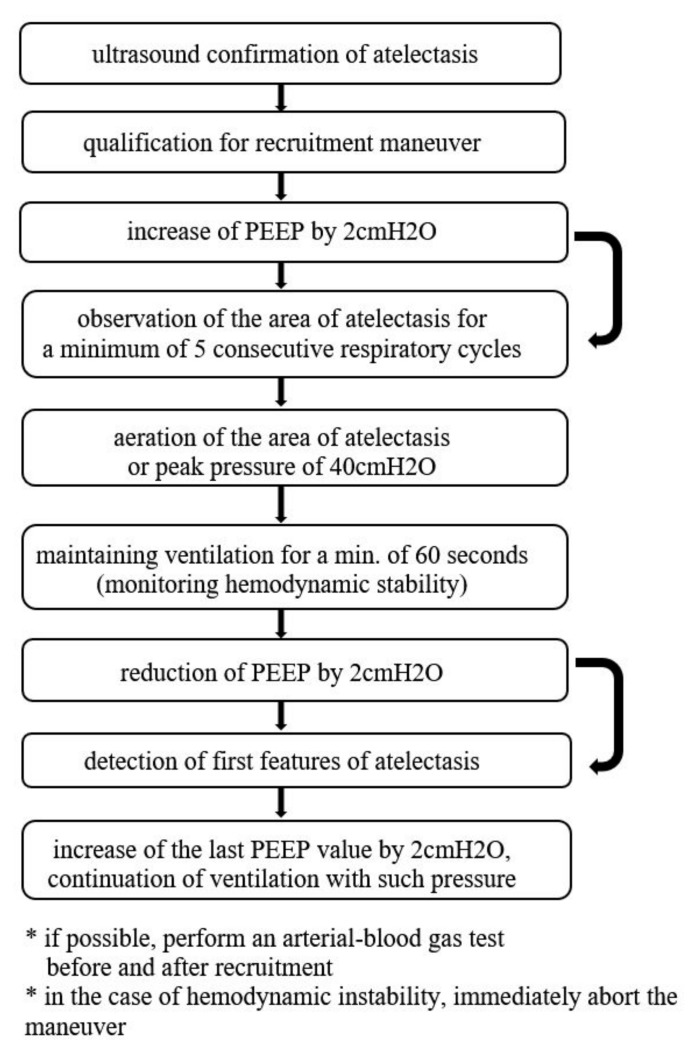
Recruitment maneuver algorithm employed in the study (modified Tusman’s protocol [22]).

The suggested recruitment method involved an incremental increase in positive end-expiratory pressure (PEEP) with simultaneous continuous ultrasound assessments. During the entire procedure, the transducer head was placed over one point of the chest, selected by the operator, where the area of atelectasis was detected intraoperatively. After each increase in the PEEP value by 2cmH_2_O, the area of atelectasis was observed for a minimum of 5 consecutive respiratory cycles. The PEEP value was increased until the aeration of the area of atelectasis (max. value of 19cmH_2_O, which resulted from the limitations of the anesthesia machine) or until peak pressure values of 40cmH_2_O were obtained. When the aim was achieved (i.e., aeration of the area of atelectasis was visualized in the ultrasound image), ventilation with patient-specific pressure was maintained for a minimum of 60 s, with a simultaneous monitoring of hemodynamic stability. Next, the PEEP was reduced by 2cmH_2_O every 5 respiratory cycles until detecting the first features of atelectasis—then the last PEEP value was increased by 2cmH_2_O and ventilation was continued at such pressure. The last ultrasound assessment was performed 2 h after extubation (Figure 2). 

### 2.7. Statistical Analysis

The collected data were analyzed statistically using IBM SPSS Statistics 25.0 software. To compare the two groups for qualitative data (nominal or categorical), Pearson’s chi-squared test was used, or Fisher’s exact test when the expected number was smaller than 5. For quantitative data, Student’s t-test was used for independent variables or Mann–Whitney U test when the numbers in the compared groups were different. To compare the results of lung ultrasound assessment at 6 points (normal vs. abnormal) during one assessment, Cochran’s Q test was employed. To establish correlations between quantitative/ordinal data, correlation analysis was performed with the use of Spearman’s rank correlation coefficient. To assess changes in compliance within the stages of assessments, repeated measures analysis of variance was performed, and to assess changes in saturation—its non-parametric equivalent—the Friedman test was used. The level of significance was α = 0.05. 

## 3. Results

### 3.1. Analysis of the Study Group and the Control Group 

The study group was composed of 100 patients. The average age of patients was about 64 years, and the average BMI value was about 28. Arterial hypertension was the most frequent chronic coexisting disease (55% of patients). In the majority of cases, surgery was elective, and its duration usually did not exceed 4 h (Table 1).

### 3.2. Preoperative Ultrasound Assessment 

In the preoperative ultrasound assessment, a normal lung image was obtained in 81 patients (81%). Eight patients (8%) had features of pulmonary congestion (bilaterally, multiple B-line artifacts in lower lung fields). The presence of atelectasis affecting areas of lung parenchyma was detected in 11 patients (11%) at this stage, in one case with the accompanying anechoic fluid in the pleural cavity. Additionally, decreased transcutaneous oxygen saturation when breathing air (93% or lower) was detected in nine patients (the lung image had features of abnormalities in six patients in this group).

### 3.3. Intraoperative Ultrasound Assessment

Perioperative atelectasis was found in ultrasound images of 87 patients (87%) in total. In 11 cases (11%), atelectasis was detected already in the preoperative assessment, in 14 patients (14%) it was visualized after 10 min of mechanical ventilation. Due to decreased values of transcutaneous oxygen saturation and/or decreased lung compliance by a minimum of 15% in relation to the initial value, ultrasound reassessment was performed in 62 patients. In all cases, areas of atelectasis were revealed. Decreased saturation, as an isolated parameter qualifying for lung ultrasound reassessment, only occurred in two cases. In 13 patients (13%), the first two ultrasound assessments did not visualize areas of atelectasis, and decreased oxygen saturation and lung compliance were not observed during anesthesia.

Eventually, 86 patients were qualified for the ultrasound-guided recruitment maneuver. One patient, despite detected atelectasis accompanied with decreased compliance, was disqualified from the recruitment maneuver due to hemodynamic instability and the necessity to administer noradrenaline infusion. In all qualified patients (100%), areas of atelectasis were visualized. Decrease in saturation occurred in 12 patients (14%), and significant decrease in compliance in 77 patients (89.5%).

### 3.4. Effect of Ultrasound-Guided Recruitment Maneuver 

**Figure 3:**
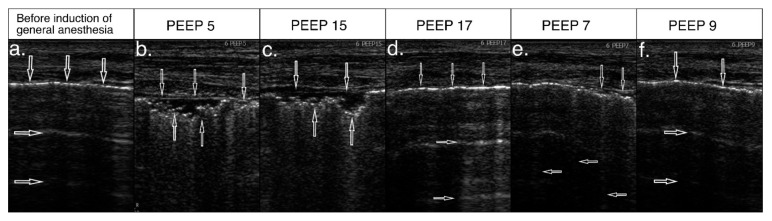
Recruitment process with a positive ultrasound effect. (**a**) Lung ultrasound (LUS) image before anesthesia—normal, hyperechoic pleural line (↓) and A-line artifacts (→), normal image; (**b**) control assessment during anesthesia, at positive end-expiratory pressure (PEEP) 5cmH_2_O—abnormal, fragmented pleural line (↓), subpleural consolidation (↑), A-line artifacts not visible, image characteristic for atelectasis; (**c**) when increasing PEEP to 15cmH_2_O —ultrasound features of atelectasis persist; (**d**) when achieving PEEP value of 17cmH_2_O—normal, hyperechoic pleural line (↓) and A-line artifact (→) visible again; (**e**) when reducing pressures, PEEP 7cmH_2_O—segmental disturbances in the pleural line continuity (↓) visible again and vertical artifacts reappear (←)—initial image of atelectasis; (**f**) after increasing end-expiratory pressure by 2cmH_2_O, disturbances in lung aeration reversed and normal pleural line and A-line artifacts were visualized.

**Figure 4:**
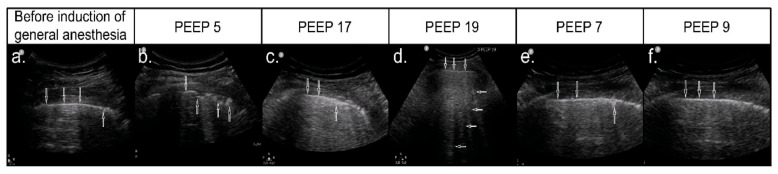
Recruitment process with an incomplete ultrasound effect. (**a**) LUS image before anesthesia—normal, hyperechoic pleural line (↓) and small abnormalities within the pleural line (↑) with small subpleural consolidations, as in segmental atelectasis; (**b**) control assessment after the induction of anesthesia, at PEEP 5cmH_2_O—blurred fragmented pleural line (↓) with hypoechoic subpleural consolidations (↑), image typical of atelectasis; (**c**) when increasing PEEP to 17cmH_2_O—persistent ultrasound features of atelectasis with a visible reduction in subpleural consolidations (↑); (**d**) when achieving PEEP value of 19cmH_2_O—continuous pleural line (↓) with multiple B-Line artifacts B (←); (**e**) when reducing pressures, PEEP 9cmH_2_O—segmental disturbances in the pleural line continuity (↑) visible again—initial image of atelectasis; (**f**) after increasing end-expiratory pressure by 2cmH_2_O, to 9cmH_2_O, improvement in the pleural line image and better lung aeration were achieved.

Recruitment maneuvers followed the adopted protocol. In 79 patients (91.9%), the recruitment was effective, i.e., aeration of the areas of atelectasis was achieved. Completely normal lung ultrasound images were revealed in 29 patients (33.7%) (Figure 3, Figure 4). Improvement of aeration, but with persistent interstitial syndrome, was found in 50 patients (58.1%) (Figure 4), and lack of improvement, that is persistent atelectasis, was observed in 7 patients (8.1%). In six patients (7%), mild hypotension was observed during PEEP de-escalation. Due to the clinical status of these patients, recruitment protocol was modified.

### 3.5. Postoperative Ultrasound Assessment

**Figure 5:**
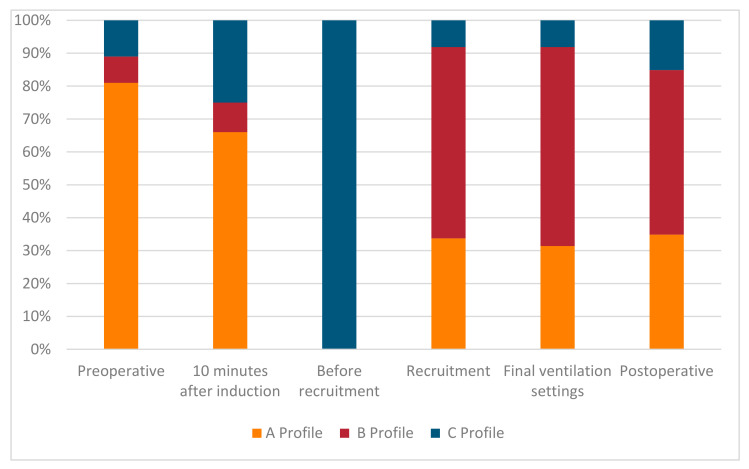
Percentage distribution of results at consecutive stages of lung ultrasound assessment.

In postoperative ultrasound assessment, the lung image was normal in 52 patients (52%), in 43 patients (43%) interstitial syndrome was visible, and atelectasis was detected in only 13 patients (13%). The percentage distribution of ultrasound assessment results at consecutive stages of the procedure are presented in Figure 5.

The localization of the ultrasound-detected areas of atelectasis during the entire procedure was analyzed. Statistically, significantly more frequent abnormal results (areas of atelectasis) were found bilaterally in the lower fields of the lateral chest (assessment points 3 and 6) at each stage of the assessment as compared to the remaining points (*p* ≤ 0.001) (Table 2). Consequently, it may be assumed that points 3 and 6 were crucial because they indicated the largest percentage of examined patients with abnormal results. 

### 3.6. Analysis of Intraoperative Mechanical Ventilation Parameters

Next, mechanical ventilation parameters were analyzed. The following data were considered: peak pressure, PEEP, lung compliance, and, additionally, oxygen saturation. Detailed results are presented in Table 3. 

Statistical analyses revealed that the mean PEEP at which atelectasis reversed in the ultrasound image was 17cmH_2_O. In the case of one patient (1.1%), sufficient PEEP resulting in the reduction in atelectasis was 9cmH_2_O, for three patients (3.4%) the PEEP value was 11cmH_2_O, and for nine patients (10.4%) it was 13cmH_2_O. The mean end-expiratory pressure preventing the alveoli collapse was 9cmH_2_O (the minimal value: 5cmH_2_O, the maximum value: 11cmH_2_O). During the performed recruitment process, no patient achieved peak pressure higher than 34cmH_2_O, and mean peak pressure was 28cmH_2_O. The difference between peak pressure and the value of end-expiratory pressure remained low during the entire recruitment process, and in the final stage it was lower than at the beginning of anesthesia.

### 3.7. Analysis of Transcutaneous Oxygen Saturation in the Perioperative Period

The analysis revealed that the saturation level at the preoperative assessment was significantly lower than during other measurements (*p* < 0.001). In the 2 h postoperative period, saturation levels did not decrease, including those patients who had abnormal saturation before surgery. In the entire study group, SaO_2_ ranged between 97 and 100%.

### 3.8. Analysis of Changes in Lung Compliance in the Perioperative Period

Repeated measures analysis of variance was performed to establish changes in compliance at consecutive stages of the procedure. Statistically significant differences were revealed between all taken measurements (*p* < 0.001). The lowest compliance level occurred before recruitment (*M* = 35.81; *SE* = 0.94), and the highest in the measurement taken after recruitment maneuvers (*M* = 49.91; *SE* = 1.33). 

## 4. Discussion

Recruitment maneuvers are a routine intervention procedure in mechanically ventilated patients. Irrespective of whether they are administered for patients undergoing surgery or patients with acute respiratory distress syndrome (ARDS) treated at the ICU, they require monitoring to assess their effectiveness. Apart from clinical monitoring (e.g., assessment of lung compliance dynamics, assessment of arterial blood gas), it is also possible to assess the effectiveness of the performed maneuvers using ultrasound [23]. Numerous publications concerning the employment of computed tomography (CT) [24,25] and electrical impedance tomography [12,26,27] have been published. CT requires the patient to be transported to the radiology unit, which is not always possible due to the patient’s status, and is actually impossible for patients for whom recruitment is performed intraoperatively. Despite common access to ultrasound devices in operating rooms and ICUs, the number of publications devoted to the use of ultrasonography in monitoring recruitment maneuvers is scarce and studies refer mostly to patients with ARDS [28,29,30]. In a healthy lung, in a dynamic ultrasound image, an aerated lung is characterized by the normal pleural line, mirror-image artifact and A-line artifacts with simultaneously preserved lung sliding [31]. Along with the reduction in aeration, single, and with the exacerbation of atelectasis, multiple overlapping B-lines appear. The next stage is the appearance of subpleural consolidations with a static air bronchogram or without bronchogram, with frequent B-lines coexisting marginally. When the aeration of pulmonary alveoli improves, the changes are observed in the reverse order: the initial subpleural consolidations will turn into B-line artifacts, and with further improvement—it is possible to obtain the normal lung image, i.e., A-line artifacts [31,32,33,34,35].

From the publications discussing this topic, Tusman’s paper [22] merits attention as he proposes and justifies the use of ultrasound during recruitment. The algorithm suggested by him, after modification, was implemented in this study. The study published by Généreux [36] reported that areas of atelectasis were significantly less frequently visualized in ultrasound in patients who underwent recruitment maneuvers; however, this effect disappeared after extubation. In our study, the permanent effect of improved lung aeration was achieved, and the recruited status did not lessen after the discontinuation of mechanical ventilation. We associate this with retaining patient-specific PEEP after the completed recruitment maneuver. We believe that the continuation of ventilation with individually determined end-expiratory pressure level prevents the worsening of lung aeration and improves the final outcome of the procedure. The study published by Song [37] is interesting in this context as it discusses the employment of lung ultrasound in preventing anesthesia-induced atelectasis in infants. It reported that the PEEP level of 5cmH_2_O did not prevent the development of atelectasis. We obtained similar results in our study—at the initial PEEP of 5cmH_2_O, in 87% of patients we detected subpleural areas of atelectasis. The mean pressure that prevented disturbances in aeration was 9cmH_2_O. Considering the specificity of mechanical ventilation in the pediatric population and significant differences in lung compliance in children, these results, we believe, are not directly comparable. 

The main aim of this study was to determine whether the suggested recruitment method with a simultaneous ultrasound assessment may lead to the reduction in mechanical ventilation pressures owing to the patient-based adjustment of the therapy. We revealed that in 91.9% of patients it was possible to recruit atelectasis successfully with the mean peak pressure of 29cmH_2_O and the mean PEEP of 17cmH_2_O. The achieved pressure values are significantly lower as compared to non-customized therapy, which reduces the risk of hyperinflation and other complications. In seven patients (8.1%) the reduction in atelectasis, as observed in the ultrasound image, was not successful. We suppose that this may be associated with additional overlapping pathologies in these patients, e.g., heart failure and pulmonary congestion. This issue requires further research—extending the assessment to include echocardiographic projections and, additionally, the assessment of the inferior vena cava [38,39,40,41,42,43].

An important issue observed in our study is that abnormalities in lung ultrasound images were found preoperatively in as many as 19 patients (19%); 5 of them underwent emergency surgery, and 14 had elective surgery. Patients who qualified for elective surgeries did not present with dyspnea and symptoms of decompensated heart failure. This observation confirms that lung ultrasound is a diagnostic tool that facilitates the detection of lung pathologies at an early stage, before overt clinical symptoms appear [31,32,33]. Abnormalities detected preoperatively impacted the activities undertaken intra- and post-operatively, mostly fluid therapy, decisions concerning the prolonged monitoring of the patient’s status, and the employment of high-flow nasal cannula and respiratory rehabilitation. In our view, the suggested procedure reduces the risk of postoperative respiratory complications. However, it is most beneficial for patients with comorbidities. In the available literature, there are reports that do not confirm the effectiveness of recruitment maneuvers in the context of reducing the risk of postoperative complications [44,45,46], Yet, these studies were not based on ultrasound monitoring.

Irrespective of the recruitment method, this procedure is associated with the risk of complications (e.g., barotrauma, volutrauma, hemodynamic destabilization). In our study, we did not find any significant clinical complications arising from alveolar recruitment. The proposed method assumes a gradual and slow increase in the PEEP level, facilitating the adaptation of the circulatory system to pressure changes in the chest. Moreover, maneuvers were performed in patients with stabilized intravascular volume, which significantly reduced the risk of hypotension. During classic intraoperative monitoring, including ultrasonography, we are not able to detect the risk of barotrauna and volutrauma. To this end, it is necessary to measure transpulmonary pressure [47,48].

In our study, we have revealed the positive impact of the employed method, but this method has, however, some limitations. The interpretation of the ultrasound image is largely dependent on the operator. Consequently, in order to increase the reliability of our results, all ultrasound examinations in our study were performed by one person, experienced in lung ultrasound assessment. It would be optimal if ultrasound images were assessed by two operators independently, taking into account the degree of agreement and consistency between the results. However, the limitations imposed by performing the examinations in the operating room make it impossible to implement such a solution. Another important limitation, in our view, is the fact that lung ultrasound does not detect hyperinflation that may occur during recruitment, definitely an unwanted phenomenon. Ultrasound images of normally aerated lungs and excessively aerated lungs will be identical. Considering that owing to the customization of the recruitment process, quite low peak pressures were obtained (on average 29cmH_2_O), the risk of hyperinflation seems lower than in traditional recruitment maneuvers, where often pressures of approximately 40cmH_2_O are used. B-lines in lung ultrasound image suggest pathologies involving the interstitium. This may indicate progressing atelectasis, but the appearance of B-lines artifacts may also result from intraoperative coexisting circulatory insufficiency or hypervolemia. It is not possible to differentiate the etiology of B-lines in ultrasound. The type of surgery, the position of the patient, and the type of surgical draping may make the ultrasound examination difficult to perform effectively for the operator. This study concerns, for the most part, patients who underwent elective surgeries and whose initial status was generally good. The results cannot be referred to patients with severe lung diseases, respiratory failure and ARDS.

## 5. Conclusions

Ultrasound-guided recruitment maneuvers facilitate the procedure customization, thus allowing for the reduction in ventilation pressures required to aerate the areas of intraoperative atelectasis, simultaneously reducing the risk of complications resulting from the procedure. The described method allows for the individual patient-based adjustment of the PEEP value that prevents atelectasis. The suggested protocol may be particularly beneficial for patients with a high risk of postoperative respiratory complications.

## Figures and Tables

**Table 1:** Clinical characteristics of study group.

Variable	Data (*n* = 100)
Gender *n* (%)	
Females	66 (66.0)
Males	34 (34.0)
Age *M*(*SD*)	63.90 (11.34)
BMI *M*(*SD*)	28.31 (5.08)
ASA Score *n* (%)	
1	1 (1.0)
2	27 (27.0)
3	66 (66.0)
4	6 (6.0)
MRC Score *n* (%)	
0	49 (49.0)
1	40 (40.0)
2	11 (11.0)
Coexisting chronic disease *n* (%)	
Hypertension	55 (55.0)
Ischemic heart disease	13 (13.0)
COPD	1 (1.0)
Asthma	6 (6.0)
Diabetes	24 (24.0)
Atherosclerosis	12 (12.0)
Type of surgery *n* (%)	
Elective	90 (90.0)
Emergency	10 (10.0)
Method *n* (%)	
Classic	82 (82.0)
Laparoscopy	18 (18.0)
Surgery duration *n* (%)	
<2 h	45 (45.0)
2–4 h	44 (44.0)
>4 h	11 (11.0)

**Table 2:** Frequency of atelectasis depending on localization.

Assessment	Atelectasis (N) *n* (%)							
	1	2	3	4	5	6	Q(5)	*p*
preoperative	1%	3%	9%	1%	3%	9%	30.59	<0.001
10 min. after induction	1%	3%	20%	1%	5%	23%	85.17	<0.001
before recruitment	2%	10%	92%	2%	13%	92%	343.00	<0.001
recruitment	2%	2%	6%	2%	2%	7%	21.15	0.001
final PEEP settings	2%	2%	6%	2%	2%	7%	21.15	0.001
2 h after extubation	2%	2%	10%	2%	2%	13%	40.33	<0.001

Legend. 1—right side—upper field; 2—right side—middle field; 3—right side—lower field; 4—left side—upper field; 5—left side—middle field; 6—left side—lower field. *Q*—Cochran’s *Q* test value; *p*—test probability.

**Table 3:** Basic statistics for peak pressure, PEEP, saturation and compliance at consecutive stages of the procedure.

Assessment	Me	IQR	Min.	Max.
preoperatively				
Saturation	96.00	2.00	88.00	99.00
10 min. after induction				
Saturation	99.00	1.00	92.00	100.00
Peak pressure	15.50	3.00	12.00	25.00
Compliance	42.00	11.00	20.00	70.00
PEEP	5.00	0.00	5.00	5.00
before recruitment				
Saturation	99.00	2.00	92.00	100.00
Peak pressure	18.00	3.00	12.00	26.00
Compliance	34.00	11.25	19.00	60.00
PEEP	5.00	0.00	5.00	5.00
recruitment				
Saturation	99.00	0.00	96.00	100.00
Peak pressure	29.00	4.00	19.00	34.00
PEEP	17.00	2.50	9.00	19.00
final ventilation settings				
Saturation	99.00	0.00	98.00	100.00
Peak pressure	18.00	4.00	13.00	26.00
Compliance	47.00	15.50	29.00	89.00
PEEP	9.00	2.00	5.00	11.00
2 h after extubation				
Saturation	99.00	0.00	97.00	100.00

Legend. Me—median; IQR—interquartile range; Min.—minimum value; Max.—maximum value.
